# Histone Modification Pathways Suppressing Cryptic Transcription

**DOI:** 10.3390/epigenomes8040042

**Published:** 2024-11-12

**Authors:** Hong-Yeoul Ryu

**Affiliations:** 1 KNU G-LAMP Project Group, KNU Institute of Basic Sciences, Kyungpook National University, Daegu 41566, Republic of Korea; rhr4757@knu.ac.kr; Tel.: +82-53-950-6352; 2 BK21 FOUR KNU Creative BioResearch Group, School of Life Sciences, College of Natural Sciences, Kyungpook National University, Daegu 41566, Republic of Korea

**Keywords:** cryptic transcription, H3K36 methylation, H3K4 methylation, Rpd3S HDAC complex, Set3 HDAC complex

## Abstract

Cryptic transcription refers to the unintended expression of non-canonical sites within the genome, producing aberrant RNA and proteins that may disrupt cellular functions. In this opinion piece, I will explore the role of histone modifications in modulating cryptic transcription and its implications for gene expression and cellular integrity, particularly with a focus on H3K36 and H3K4 methylation marks. H3K36 tri-methylation plays a crucial role in maintaining chromatin integrity by facilitating the recruitment of the Rpd3S histone deacetylase (HDAC) complex, which helps restore closed chromatin states following transcription and prevents cryptic initiation within gene bodies. In parallel, crosstalk between H3K4 di-methylation and histone ubiquitylation and sumoylation is critical for recruiting the Set3 HDAC complex, which maintains low histone acetylation levels in gene bodies and further suppresses cryptic transcription. Therefore, by elucidating these regulatory mechanisms, this opinion highlights the intricate interplay of histone modifications in preserving transcriptional fidelity and suggests potential pathways for future research to develop novel therapies for age-related disorders and other diseases associated with dysregulated gene expression.

## 1. Introduction

Chromatin is built from nucleosomes, dynamically regulated multiprotein complexes that serve as genomic DNA scaffolds [1]. Each nucleosome comprises 145 to 147 DNA base pairs coiled around a histone protein octamer, consisting of two copies of each histone H2A, H2B, H3, and H4, which play a role in higher-order chromatin compaction. This nucleosome structure both facilitates DNA packing within the nucleus and restricts access to large portions of the genome, significantly impacting gene expression regulation. Therefore, the transcription process, which produces RNA from DNA, relies on proteins, such as transcription activators and chromatin remodeling factors, to ensure proper temporal and spatial access to specific DNA regions [2]. Thus, errors in this process can potentially lead to severe consequences for various cellular functions.

Cryptic transcription refers to the process of transcription occurring at unexpected or non-canonical sites within the genome, involving the transcription of DNA regions that are typically silent or not intended to be expressed under normal conditions [3]. This phenomenon can lead to a waste of cellular energy and resources needed for normal RNA synthesis, resulting in the production of aberrant RNA molecules that may generate non-functional or harmful proteins, create RNA interference, or affect the stability and processing of other RNA molecules [3]. Therefore, cells employ diverse mechanisms, such as histone modification, chromatin remodeling, or transcription termination-coupled RNA decay, to prevent the transcriptional machinery from accessing cryptic sites [4].

Histone proteins undergo various post-translational modifications, such as methylation of arginine (R), phosphorylation of serine (S) and threonine (T), and several other lysine (K) residue modifications, including acetylation (ac), methylation (me), ubiquitylation (ub), sumoylation, biotinylation, and ADP-ribosylation [5,6,7]. These modifications are important for regulating chromatin structure and its accessibility, determining whether chromatin is in an open or closed state. The formation of open chromatin at unintended or cryptic sites can lead to increased cryptic transcription, which is closely regulated by histone modifications [3,8]. Specifically, histone modifications associated with open chromatin, such as acetylation (e.g., H3K27ac) and certain methylations (e.g., H3K4me3), can inadvertently reveal cryptic transcription start sites [3]. These changes reduce nucleosome density and facilitate the binding of transcription factors and RNA polymerase (Pol), potentially resulting in cryptic transcription. Inversely, histone modifications can function as crucial factors in preventing cryptic transcription [3,8]. Therefore, this opinion provides an overview of the currently known pathways in which histone modifications occur to restrict cryptic transcription.

## 2. Chromatin Structure and Cryptic Transcription

The chromatin structure must be dynamically altered and reorganized during transcription elongation, as failure to reassemble the chromatin structure properly can expose cryptic promoter elements, leading to the initiation of aberrant transcription from intragenic regions in either a TATA-dependent or TATA-independent manner [3,9]. Transcription elongation factors are critical in maintaining transcription fidelity by ensuring proper elongation, chromatin structure, and RNA processing. Their role in managing these processes helps suppress cryptic transcription initiation, thereby maintaining gene expression accuracy and cellular function. In *S. cerevisiae*, among such factors involved in blocking cryptic transcription, Spt6 is highlighted as a key player since it can facilitate efficient transcription elongation and plays a vital role in stabilizing chromatin structure, further contributing to suppressing cryptic transcription [10,11]. Other elongation and chromatin factors, including Spt16 (a FACT complex subunit), Spt10, Spt2, Hir1, Elf1, and histone H3, also play a role in this regulatory control [10,12,13]. By preserving a well-defined chromatin structure over transcribed regions, RNA Pol II and its associated factors prevent inappropriate initiation, which could disrupt normal gene expression. In mammals, it has been shown that perturbations that alter the post-transcription chromatin state also promote cryptic transcription, which has also been noted in *S. cerevisiae* [14,15,16,17].

## 3. H3K36 Methylation and Cryptic Transcription

**Figure 1:**
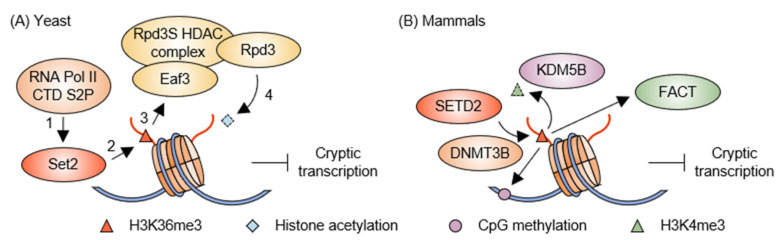
Suppression of cryptic transcription by the H3K36 methylation pathway. (**A**) In yeast, the recruitment of Set2 depends on the state of CTD S2P on RNA Pol II. The Eaf3 subunit in the Rpd3S HDAC complex recognizes Set2-mediated H3K36me3 and recruits the complex to the gene bodies, effectively suppressing cryptic transcription. (**B**) In mammals, H3K36me3 is co-transcriptionally added by SETD2 and serves as a scaffold to recruit the DNMT3B DNA methyltransferase, KDM5B H3K4 demethylase, and FACT histone chaperone complex. These processes work together to repress cryptic transcription.

Histone acetylation enhances transcriptional elongation by loosening the chromatin structure, allowing RNA Pol II easier access to DNA for efficient transcription [18]. Histone acetylation should be erased following transcriptional elongation to prevent cryptic transcription within the gene body regions [19]. Yeast Rpd3 is the histone deacetylase (HDAC) enzyme with the best-established role in inhibiting cryptic transcriptional initiation. The Rpd3 is part of two distinct multi-subunit complexes, Rpd3L and Rpd3S, which are recruited to different chromosome regions and perform distinct functions in gene regulation [20]. Furthermore, Set2 histone methyltransferase-mediated H3K36 tri-methylation (me3) acts as an epigenetic mark for Rpd3S complex loading, with the Eaf3 subunit recognizing H3K36me3, resulting in histone deacetylation of the gene bodies [21,22,23,24]. According to previous studies in *S. cerevisiae*, this Set2–Rpd3S pathway governs cryptic initiation in ~30% of yeast genes, suggesting that the H3K36me3-originated chromatin modifications are important for maintaining genome integrity [25,26] (Figure 1A). The Rpd3S complex features the unique assembly of Eaf3 and Rco1 subunits with Rpd3 and Sin3, facilitating multivalent interactions necessary for efficient deacetylation [27,28]. Sin3 and Rco1 coordinate binding with nucleosomes, where Rco1′s PHD1 domain targets unmodified H3K4 and Eaf3 recognizes H3K36me3 marks, effectively preventing cryptic transcription [27,28]. Additionally, a second set of Eaf3 and Rco1 subunits interacts with neighboring nucleosomes, indicating a dynamic mechanism for chromatin engagement and regulation [27].

The Set2–Rpd3S pathway is also intricately regulated by post-translational modifications of the RNA Pol II carboxy-terminal domain (CTD), characterized by conserved heptapeptide repeats (Y^1^-S^2^-P^3^-T⁴-S⁵-P⁶-S⁷) [29,30]. In eukaryotic cells, the CTD undergo various modifications, particularly phosphorylation [31,32]. Among these modifications, the phosphorylation of serines S2 and S5 (S2P and S5P) is highly significant. S5P levels peak as transcription moves from initiation to early elongation, while S2P levels increase during productive elongation, reaching their highest point near the polyadenylation signal. A loss of CTD phosphorylation results in the dissociation of Set2 from RNA Pol II [33]. Notably, in *S. pombe*, the absence of S2P leads to a genome-wide increase in antisense transcription, which correlates with enhanced histone acetylation within gene bodies [34]. Thus, CTD S2P is essential for regulating the Set2–Rpd3S pathway, thereby preventing the initiation of cryptic intragenic transcription.

In mammals, the presence of H3K36me3 within gene bodies plays a critical role in repressing abnormal intragenic transcription. This regulation occurs through the recruitment of factors such as the DNMT3B DNA methyltransferase, KDM5B H3K4 demethylase, and the FACT complex, which acts as a chaperone to replenish nucleosomes following RNA Pol II passage [35]. KDM5B specifically removes H3K4me3 marks—markers of active transcription—from the gene body [15], while DNMT3B establishes new CpG methylation—markers of repressive transcription—within the gene [17]. Consequently, although transcription elongation leads to chromatin accessibility, those additional histone modifications help organize various complexes to restore chromatin to a tighter closed structure after Pol II activity. When this regulatory process is disrupted, actively transcribed genes may fail to revert to their closed chromatin state, resulting in increased cryptic intragenic transcription. For instance, the loss of *SETD2*, an enzyme responsible for H3K36me3 modification, is associated with heightened levels of cryptic transcription in both human mesenchymal stem cells and murine embryonic stem cells (ESCs) [16,17]. The loss of *SETD2* in ESCs disrupts the intragenic recruitment of DNMT3B, resulting in increased cryptic transcription [17]. However, other studies have shown that an inducible double knockout of *DNMT3A* and *DNMT3B*, conditions that inhibit DNA methylation, and an inducible triple knockout of *DNMT1*, *DNMT3A*, and *DNMT3B* do not increase cryptic transcription levels [36,37]. The discrepancy in these findings may result from differences in the timing of the DNA methylation loss or changes in methylation patterns when various methyltransferases are affected [38]. Moreover, the depletion of *KDM5B*, which is recruited to transcribed genes by H3K36me3, also leads to an increase in intragenic cryptic transcription [15]. Since CpG methylation is typically associated with gene promoter silencing, a decrease in this modification following the loss of *DNMT3B* would likely diminish the inhibition of cryptic promoters in the gene. Similarly, increased H3K4me3 levels resulting from the loss of *KDM5B* encourage an active chromatin state, which resembles a promoter within the gene body. As both DNMT3B and KDM5B are localized to chromatin via H3K36me3 at transcribed gene bodies, which SETD2 confers, the absence of *SETD2* leads to downstream effects similar to those observed following *DNMT3B* or *KDM5B* loss while possibly maintaining the chromatin in a relatively nucleosome-depleted condition due to decreased FACT recruitment (Figure 1B).

## 4. H3K4 Methylation and Cryptic Transcription

The interplay between post-translational modifications of histone proteins plays a crucial role in regulating chromatin structure and gene expression [39]. A prominent example of this interaction is H2BK123 mono-ubiquitination (H2Bub), which facilitates the unidirectional methylation of K4 and K79 on histone H3, mediated by Set1 and Dot1 in *S. cerevisiae* [40]. The resulting H2Bub-dependent methylation of H3K4 displays a characteristic gradient pattern: H3K4me3 occurs near gene promoters, H3K4 di-methylation (me2) is found immediately downstream, and H3K4 mono-methylation (me1) is observed in more distal regions [41,42]. This gradient is influenced by the period that Set1 remains associated with RNA Pol II during successive transcription [43]. While the function of H3K4me3 in transcription has been well documented [44], understanding the role of H3K4me2 remains limited.

Current research indicates that H3K4me2 uniquely affects the transcription cycle [45]. When Set1 is expressed without the RNA recognition motif (RRM), leading to the absence of H3K4me3 while leaving H3K4me2 intact, there is no significant increase in histone acetylation in the 5′ transcribed regions [45,46]. This suggests that H3K4me2 alone can inhibit histone acetylation within the 5′ open reading frame (ORF). In addition, among various candidate proteins, including those containing the PHD domain, which binds to methylated H3K4, Set3 shows a preference for H3K4me2 peptides [47]. This interaction allows H3K4me2 to influence the recruitment of the Set3 complex, which contains two active HDAC subunits, Hos2 and Hst1, to the chromatin [45]. The absence of these subunits, alongside additional proteins, such as Sif2 (WD40 protein) and Snt1 (SANT domain protein), associated with the Set3 complex, results in hyper-histone acetylation at the 5′ ORF sites [45]. Collectively, histone deacetylation, mediated by the Set3 HDAC complex, in these regions acts to inhibit the cryptic initiation of both sense and antisense transcription within the ORFs [48]. In summary, these findings suggest that H2Bub and the subsequent H3K4me2-mediated deacetylation play critical roles in preserving transcriptional accuracy by preventing the initiation of cryptic transcription.

**Figure 2:**
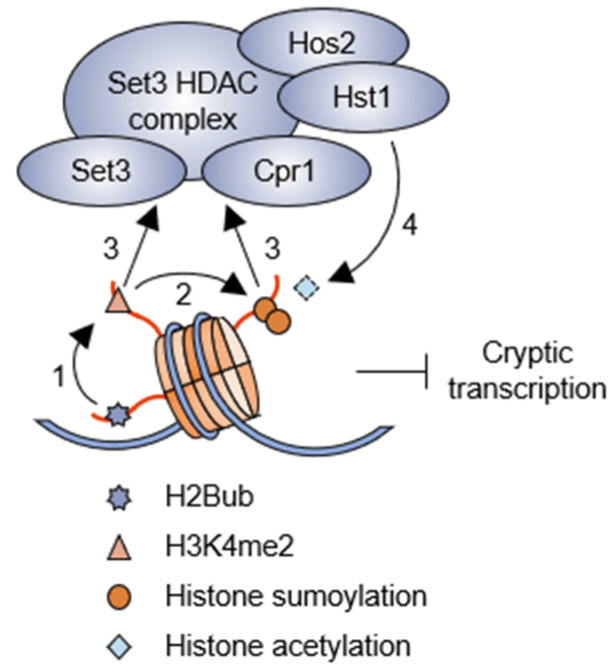
Suppression of cryptic transcription by the H3K4 methylation crosstalk pathway. H2Bub promotes H3K4me2, which subsequently leads to histone sumoylation in gene body regions. Both H3K4me2 and histone sumoylation provide distinct binding platforms for Set3 and Cpr1 within the Set3 HDAC complex, including two HDAC enzymes, Hst1 and Hos2, to suppress cryptic transcription. The numbers in the model indicate the order in which the reactions occur in the pathway.

Recent studies have shown that histone sumoylation is also intricately linked to the H2Bub-mediated H3K4 methylation pathway [49,50,51]. Histones are well-known targets for small ubiquitin-like modifier (SUMO) modifications, a process that involves multiple enzymes: the heterodimer Aos1/Uba2 (referred to as SAE1/SAE2 in mammals), the SUMO-activating enzyme (E1), the Ubc9 SUMO-conjugating enzyme (E2), and several distinct SUMO ligases (E3s) [49,52,53,54]. Similarly to the influence of H2Bub on histone sumoylation, H3K4me2 is essential for initiating the sumoylation process, whereas H3K4me3 does not play a role, thus indicating a unidirectional pathway [50]. Furthermore, a mutation in histone H2B that leads to compromised sumoylation disrupts the binding of Set3 complex subunits, Set3 and Hos2, to their target genes. This disruption is associated with increased histone acetylation levels and displays significant sensitivity to 6-azauracil, which is commonly used as an indicator of transcription elongation [55], at 34 °C [42]. Cpr1, another Set3 complex subunit, recognizes SUMO-modified histones through its SUMO-interacting motif, facilitating the recruitment of the Set3 complex to nucleosome sites [15]. Notably, the H2B mutation also impairs the ability of the Set3 complex to associate with noncoding RNA (ncRNA) genes, in addition to protein-coding genes, resulting in a marked increase in the transcription of ncRNAs from internal regions within ORFs [51]. Thus, an elaborate histone modification network involving the consecutive ubiquitination, methylation, sumoylation, and deacetylation of histones collectively promotes transcriptional elongation by suppressing cryptic intragenic transcription initiation (Figure 2).

## 5. Conclusions and Future Directions

Preventing cryptic transcription is crucial for maintaining proper gene expression and cellular function, as evidenced by the complex interplay of histone modifications and chromatin dynamics. The Rpd3S HDAC complex plays a pivotal role in regulating this interplay by using H3K36me3, in particular, to restore closed chromatin states after transcription. Disruptions in this pathway lead to increased cryptic transcription, further underscoring the importance of these modifications for genomic integrity [21,22,24,26]. Similarly, the Set3 HDAC complex highlights the significance of H2Bub, H3K4me2, and histone sumoylation in suppressing cryptic transcription through histone deacetylation, further illustrating a multilayered regulatory approach [8,49]. Overall, the intricate network of histone modifications and regulatory complexes highlights the complexity of transcriptional regulation and suggests areas for future research, particularly focusing on the dynamic nature of these modifications and their context-specific impacts on gene expression.

The increase in cryptic transcription can inadvertently enhance or suppress the expression of specific genes. This abnormal gene expression may be linked to various diseases, particularly concerning aging, with recent reports highlighting these associations [56,57]. Cryptic transcription is elevated in yeast and nematodes alongside aging, and reducing cryptic transcription has been shown to extend the lifespan of yeast [58,59,60,61,62]; similar findings have also been observed in mammals [19]. Additionally, elevated levels of cryptic transcription in aged mammalian stem cells are marked by regions exhibiting a unique chromatin signature that includes decreased H3K36me3 and increased H3K4me1, H3K4me3, and histone acetylation with age [63]. Therefore, a more thorough characterization of how epigenetic regulation influences the monitoring mechanisms of spurious transcription may provide a promising pathway for future research to develop new therapies for various disorders, including those related to aging.

## Data Availability

Not applicable.
